# Case Report: Interdisciplinary approach to managing behavioural and psychological symptoms of dementia and delirium in acute care

**DOI:** 10.3389/frdem.2026.1740269

**Published:** 2026-02-13

**Authors:** Andrea Staglianò, Sara Visconti, Roberto Lucifora, Marta Aber Rizzo, Elena Page, Maria Cristina Ferrara, Elena Pinardi, Beatrice Tonus, Annalisa Sironi, Flavia Sandi, Alberto Finazzi, Chukwuma Okoye, Paolo Mazzola, Giuseppe Bellelli

**Affiliations:** 1 Acute Geriatrics Unit, Fondazione IRCCS San Gerardo dei Tintori, Monza, Italy; 2 School of Medicine and Surgery, University of Genoa, Genoa, Italy; 3 Centro Studi per la Medicina della Complessità e Cure Palliative “Virgilio Floriani”, Milan, Italy; 4 School of Medicine and Surgery, University of Milano-Bicocca, Milan, Italy; 5 Aging Research Center, Department of Neurobiology, Care Sciences and Society, Karolinska Institutet and Stockholm University, Stockholm, Sweden

**Keywords:** acute care, BPSD, case report, dementia, multidisciplinary management

## Abstract

Behavioural and Psychological Symptoms of Dementia (BPSD) and delirium are common in acute hospital settings. Although non-pharmacological approaches are recommended as first-line interventions, BPSD and delirium are still often managed with physical restraints and psychotropic medications, which may prolong hospital stays and increase the risk of falls, institutionalisation, and readmissions. We describe four cases of older adults with dementia admitted to an Acute Geriatric ward for acute medical conditions, each presenting with a predominant behavioural symptom: agitation, aggression, apathy, and wandering, respectively. Symptom management was based on a structured, non-pharmacological, interdisciplinary approach involving medical doctor, case manager, occupational therapist, registered nurses, healthcare support workers, and volunteers. This care model enabled the individualisation of care plans according to each patient’s needs, promoted the preservation of functional independence, reduced the use of psychotropic medications, and facilitated discharge. While these cases obviously cannot provide definitive conclusions on the efficacy of the adopted model, they suggest that multidisciplinary, integrated, non-pharmacological and pharmacological approaches to BPSD and delirium may improve patient’s outcomes and well-being in hospital settings.

## Introduction

Behavioural and Psychological Symptoms of Dementia (BPSD) are integral to the dementia syndrome and are associated with substantial patient distress, caregiver burden, and accelerated functional decline (Bessey and Walaszek, 2019; Byeon et al., 2025; Department of Health, 2019; Scottish Intercollegiate Guidelines Network (SIGN), 2019). A recent systematic review and meta-analysis estimated that up to 60% of older adults with dementia admitted to acute hospital wards experience BPSD, with rates reaching 85% depending on the assessment tool used (Anantapong et al., 2025). The most common symptoms include agitation (39%), sleep disturbances (38%), eating problems (36%), and irritability (32%) (Anantapong et al., 2025). Delirium is an acute or subacute neuropsychiatric syndrome which is usually triggered by multifactorial somatic causes. Individuals with dementia are at high risk of delirium superimposed on dementia (DSD). Evidence indicates that DSD represents over 70% of delirium cases in older adults and affects nearly half of hospitalized patients with dementia (Timmons et al., 2025).

Hospitalisation can precipitate or exacerbate BPSD and delirium through environmental unfamiliarity, strict routines, occupational deprivation, and acute medical disorders (Abbott et al., 2022). Although pharmacological management is frequently employed, it carries substantial risks in frail older adults - including falls and further cognitive or functional decline (White et al., 2017). In this context, there is growing interest in non-pharmacological approaches that address the person’s needs through environmental, relational, occupational, and psychoeducational strategies (Gitlin et al., 2021). Current best practice guidelines (Department of Health, 2019; Scottish Intercollegiate Guidelines Network (SIGN), 2019; Coin et al., 2025; Hatch et al., 2025; National Library of Medicine, 2018) recommend prioritising such interventions as first-line treatments for both BPSD and delirium, reserving pharmacological therapy for cases in which symptoms endanger the patient or others, and only after potentially reversible medical or environmental factors have been excluded.

Non-pharmacological interventions are considered effective when tailored to individual needs and delivered through a structured, interdisciplinary framework (Anantapong et al., 2025; Gitlin et al., 2021; Pozzi et al., 2024). Nevertheless, their implementation in acute hospital settings remains limited, largely due to organizational constraints and the challenges of interprofessional collaboration (Dunkle et al., 2022; Sampson et al., 2014).

We report four clinical cases of older adults with BPSD and/or DSD admitted for acute medical conditions to an Acute Geriatrics Unit (AGU) of an Italian tertiary hospital. Each case illustrates a predominant behavioural presentation - agitation, aggression, apathy, or wandering – and the potential benefits of a tailored, interdisciplinary, non-pharmacological care model addressing each patient’s individual needs.

## Case description

### Case 1: agitation

An 84-year-old man was admitted to the Emergency Department (ED) of our hospital after a horseback riding accident that caused a head injury and transient loss of consciousness. Before hospitalisation, he was independent in Basic Activities of Daily Living (B-ADL 6/6) (Katz et al., 1963), but dependent in most Instrumental Activities of Daily Living (IADL 1/5) (Lawton and Brody, 1969). His Clinical Frailty Scale (CFS) (Rockwood et al., 2005) score was 6, indicating moderate frailty. His medical history included several recent unwitnessed falls and mild dementia.

After being diagnosed with a subarachnoid haemorrhage and a humeral fracture, the patient was admitted to the Short Stay Unit (SSU). Due to psychomotor agitation, he received promazine hydrochloride (50 mg, intramuscular [IM]) and trazodone (25 mg, IM) and was physically restrained. He was subsequently transferred to the AGU. On admission, he appeared slowed, inattentive, and disoriented in time, restless and confabulating. His 4AT score (Bellelli et al., 2014) was 8/12, suggesting hyperactive DSD. On the first day, the medical doctor (MD), case manager (CM), and occupational therapist (OT) conducted a comprehensive geriatric assessment (CGA), including medical, social, and functional evaluations and risk stratification. The MD promptly identified multiple potential causes of delirium - pain, restraints, a Desault bandage, and dehydration - and initiated corrective measures. Analgesic therapy and rehydration were started, and medications were reviewed to avoid the use of neuroleptics and benzodiazepines. During the night, the patient experienced severe agitation, repeatedly leaving his bed and wandering around the ward, and removing vascular accesses and the Desault bandage. He was guided back to bed, reassured, and comforted. From the second day, an individualized care plan was implemented, involving additional team members. The CM loosened the Desault bandage, removed other physical restraints, and assisted with hygiene, supported by healthcare support workers (HCSWs). The CM also encouraged the patient’s family to participate actively in the care process. The OT provided training in BADL, reorientation exercises, and meaningful activities, including card games with his son. Additionally, he provided guidance to the patient’s son on how to further engage his father. From the third day, the patient regained sustained attention and orientation to self and space. Sleep quality improved, with restoration of a regular sleep–wake cycle using a low dose of trazodone only. As agitation resolved, the patient recovered independence in BADL, including using the toilet on his own. He was discharged home with home care services, and the caregiver received guidance on non-pharmacological BPSD management strategies.

### Case 2: aggression

An 83-year-old woman was admitted to the ED because of aggressive behaviour toward a family member and episodes of self-harm that made home management unfeasible. Before admission, she was partially dependent in B-ADL (4/6) and fully dependent in IADL (0/8). Her CFS score was 7, consistent with severe frailty. According to family reports, cognitive decline had been noted for several years.

Upon arrival to ED, the patient presented with severe agitation, verbal and physical aggression toward healthcare staff, and confabulatory speech. She was refractory to management and refused any evaluation. Physical restraints were applied, and she received multiple sedatives over a six-hour period: midazolam (5 mg, intravenous [IV]), haloperidol (4 mg, IM), chlorphenamine (10 mg, IM), delorazepam (5 mg/mL, 10 drops, orally [PO]), promazine hydrochloride (25 mg, IM), and clotiapine (40 mg, IM). She was then transferred to the AGU, where she appeared drowsy but responsive to verbal stimuli and able to answer simple questions. Her 4AT score was 6/12, suggesting DSD. After a joint assessment by the MD, CM and OT, a multidisciplinary plan was initiated, tailored to her unique needs. The MD addressed underlying delirium triggers and reviewed medications. The OT engaged the patient in recreational and reading activities, involving and instructing volunteers on how to interact, reading and annotating magazines together. The CM coordinated hygiene care with HCSWs and promoted family engagement.

Through these coordinated and tailored interventions by the multidisciplinary team, aggressive behaviours gradually subsided and resolved. The patient became calm and cooperative throughout the day. Thanks to appropriate stimulation and engagement in personalized daytime activities, her sleep improved in both quality and duration. Night awakenings were managed through social engagement and conversation by registered nurses (RNs) and HCSWs. Before discharge, she regained the ability to walk independently. Given her clinical and social profile, she was discharged to a nursing home for continued care.

### Case 3: apathy

An 82-year-old woman was admitted to the ED for clinical deterioration and persistent constipation. She lived with her husband and was assisted by two live-in caregivers providing continuous 24-h assistance. She was dependent in most B-ADL (3/6) and IADL (1/8), and her CFS score was 7 (severe frailty). Family members reported a progressive reduction in food and fluid intake over 6 months, resulting in a 10-kg weight loss.

Following a diagnosis of lower-limb erysipelas, she was admitted to the AGU. On the first day, the MD, CM, and OT jointly assessed her condition and care needs. Her clinical history revealed prominent apathy, particularly affecting eating behaviour, resulting in cachexia. The MD initiated antibiotic therapy. Repeated 4AT screening did not detect delirium at admission or during the hospital stay. The OT implemented environmental and behavioural strategies to support nutrition: offering meals outside the wardroom, adjusting meal timing, and interspersing meals with pleasant, meaningful activities. The CM contacted the family members, whose collaboration remained limited, and engaged volunteers, RNs, and HCSWs, to encourage cooperation and maintain a daily food diary (HCSWs were responsible for this task). Medications were administered alongside pleasant activities, such as crossword puzzles. Creating a calm, flexible and low-stimulation environment that accommodated the patient’s rhythms improved her cooperation with proposed treatments, and food intake. After stabilisation, given her clinical and social needs, she was discharged to a nursing home.

### Case 4: wandering

A 90-year-old man was brought to the ED by a neighbour because of generalized psychomotor slowing. He was alert but disoriented in time and space and unable to sustain attention. He was independent in B-ADL (6/6), but dependent in most IADL (1/5), with a CFS score of 6 (moderate frailty). He had a known diagnosis of dementia.

He was diagnosed with urosepsis and mild heart failure. A urinary catheter was inserted, and he was started on antibiotic and diuretic therapy. After his transfer to the SSU, he experienced severe agitation and removed his urinary catheter. He was then treated with trazodone (25 mg, IM) and haloperidol (4 mg, IM), while being restrained. Upon AGU admission, he was confused, aggressive, and agitated, with a 4AT score of 12/12 suggesting hyperactive delirium.

From the first day and after initial joint assessment by MD, OT and CM, an interdisciplinary plan was initiated. The OT adapted the environment with clear signage in the room (e.g., “bathroom,” “closet”), name tags on the room door, and a recognition cue on the patient’s clothing. Volunteers were involved in group and individual activities related to his interests (e.g., nautical magazines). Visiting hours for family members were extended in agreement with the nurse manager. The MD prescribed low-dose trazodone. These measures allowed the safe removal of restraints, reduction in agitation, and promotion of independent movement within a structured and supervised space. Before discharge, the CM trained the caregiver in non-pharmacological strategies to manage BPSD, and home care services were activated to ensure continuity of care.

## Discussion

Through this case series, we showed that a well-coordinated interdisciplinary approach can support the delivery of care to patients with BPSD and/or DSD that better preserves patient dignity and may improve treatment outcomes.

**Figure 1:**
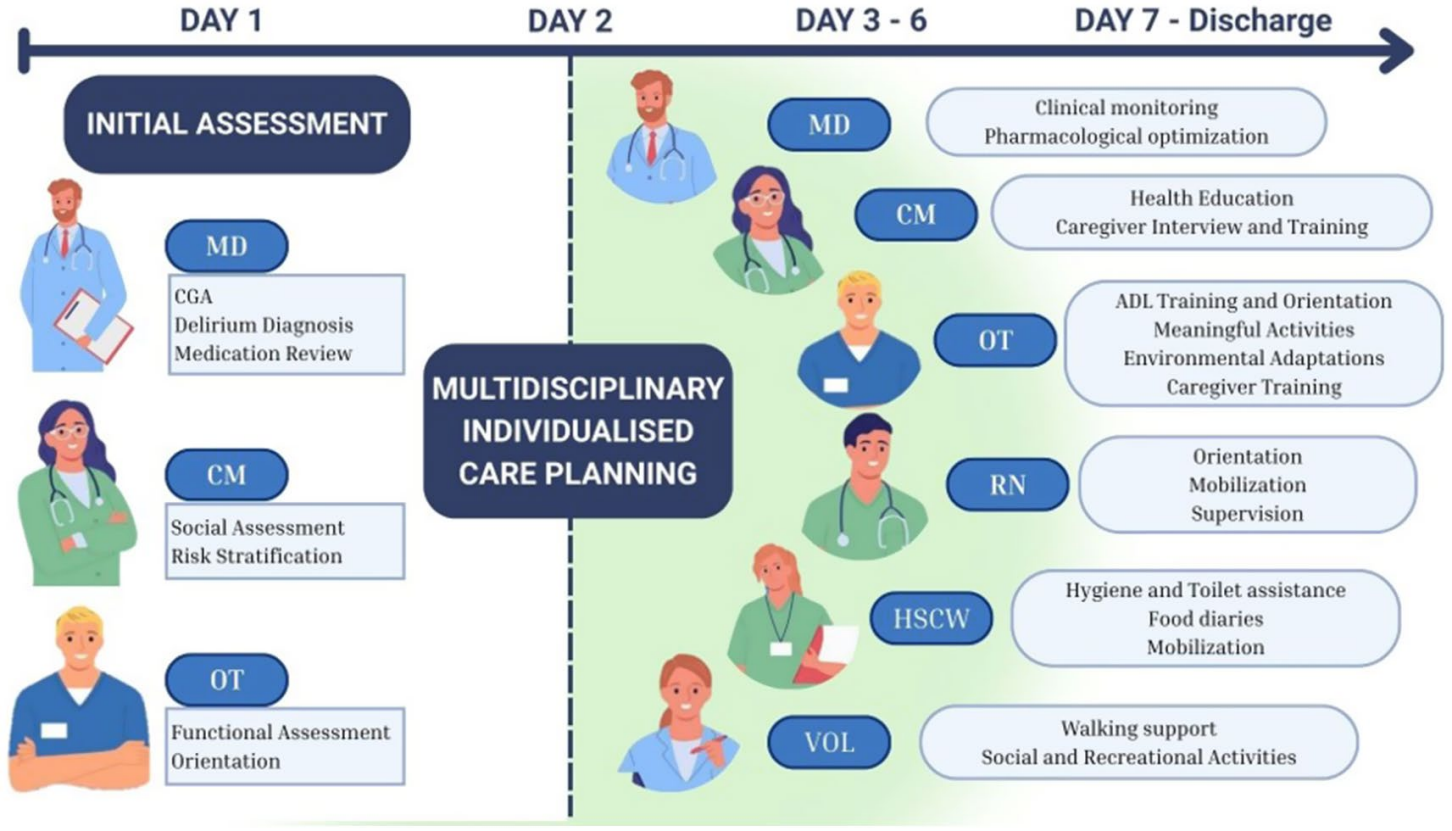
Interdisciplinary management of BPSD: roles of the healthcare professionals and timeline of intervention implementation. MD, medical doctor; CM, case manager; OT, occupational therapist; RN, registered nurse; HCSW, healthcare support worker; VOL, volunteer.

As illustrated in Figure 1, the care process begins with collaborative discussions among healthcare professionals—including MD, CM and OT—to identify individual needs and develop a shared, personalized care plan. The collaborative approach ensures that each professional subsequently involved contributes with targeted interventions aligned with the overarching care goals. Daily interdisciplinary briefings promote continuity of person-centred care, allowing ongoing strategies to be monitored enabling the team to adapt interventions to the patient’s evolving clinical and functional status. For instance, the withdrawal of physical restraints requires coordinated decision-making, close supervision, and environmental modification. This model moves beyond the mere involvement of professionals from multiple disciplines: it integrates expertise, shared decision-making, tailored interventions, and collective accountability for patient outcomes.

A key element of this model is the active involvement of caregivers, facilitated by the CM, which supports both patient well-being and continuity of care after discharge. Even when family members are not available, the CM plays a pivotal role in coordinating the multidisciplinary team and implementing alternative strategies, actively seeking flexibility and adaptability whenever required to meet the patient’s unique needs.

Intervention programmes such as the Tailored Activity Programme in OT (Gitlin et al., 2021; Pozzi et al., 2024) exemplify the principles of this model, by training caregivers to engage patients in meaningful activities while providing stress management and health education on BPSD.

Another distinctive feature is the structured involvement of trained volunteers, prepared in collaboration with the OT and briefed before each activity. Volunteer-led activities enhance patient care through social engagement and cognitive stimulation, delivered individually or in group, either in dedicated ward spaces or in the patient’s room.

Within this framework, the MD plays a central role in reviewing and optimising pharmacological therapy, including medication reconciliation and deprescribing. Psychotropic medications are reserved for cases in which BPSD and/or DSD is severe and unresponsive to non-pharmacological strategies, particularly when symptoms compromise safety or interfere with life-saving treatments. Equally essential is the early identification and correction of precipitating factors—such as dehydration, constipation, and infections (Anantapong et al., 2025; Ormseth et al., 2023)—that can trigger delirium or exacerbate BPSD and/or DSD. This step should precede any pharmacological intervention. We cannot ascertain whether some cases involved DSD or BPSD, as clinical differentiation is often challenging, particularly in advanced dementia (Timmons et al., 2025). However, management of both DSD and BPSD follows the same stepwise approach, based on non-pharmacological interventions, correction of underlying medical causes, and pharmacological treatment only when severe distress is present. The reported cases illustrate the practical organization and sequencing of these interventions.

It is likely that, in the case of the first two patients, analgesics, and in the case of the latter two, antibiotics, (Table 1) may have contributed to improve patients’ clinical outcomes.

**Table 1:** Pharmacological therapy of the four patients during their stay in the AGU.

Case No.	Predominant BPSD	Pharmacological therapy
1	Agitation	Trazodone 60 mg/mL 12 drops PO Calcium folinate 15 mg PO, Macrogol 3,350 1 sachet PO, Paracetamol 1 g IV TID, Melatonin 2 mg PO, NaCl 0.9% solution 500 mL IV, Cyanocobalamin 500 mcg IM
2	Aggression	Haloperidol 2 mg/mL 1/2 ampoule IM, Trazodone 50 mg extended release PO Paracetamol 1 g IV, Macrogol 3,350 1 sachet PO BID
3	Apathy	Haloperidol 2 mg/mL 5 drops PO, Citalopram 40 mg/mL 10 drops PO 5% Dextrose solution 500 mL IV, Ringer’s acetate solution IV, KCl 600 mg PO, Ticlopidine 250 mg PO BID, Macrogol 3,350 1 sachet PO, Amoxicillin/Clavulanate 2 + 0.2 g IV TID, Levothyroxine 100 mcg PO, Lactulose 1 tbsp PO
4	Wandering	Haloperidol 2 mg/mL IM, Trazodone 50 mg extendend release PO Furosemide 25 mg PO, Iron supplementation 500 mg IV, Saccharomyces boulardii 5 billion CFU PO, Paracetamol 1 g IV, Ringer’s acetate solution IV, Amoxicillin/Clavulanate 2 + 0.2 g IV TID, Dutasteride 0.5 mg PO, Rabeprazole 20 mg PO, Folic acid 5 mg PO, Tamsulosin 0.4 mg PO, Melatonin 2 mg PO, Finasteride 5 mg PO, KCl 600 mg PO, Clopidogrel 75 mg PO

PO, per os (orally); NaCl, sodium chloride; IV, intravenous; IM, intramuscular; KCl, potassium chloride oral supplement; tbsp, tablespoon; BID, bis in die; TID, tris in die. Psychotropic medications are indicated in blue text.

Overall, clinical observations from this case series suggest that an interdisciplinary, non-pharmacological approach may improve the management of BPSD in hospital. The healthcare team and the caregivers reported better sleep patterns, greater engagement in daily activities, improved functional independence, and overall well-being. Family members, when present, also confirmed improved patient participation and interaction.

From an organisational perspective, this interdisciplinary care model seemed to promote more flexible and coordinated care, contributing to better preservation of function, shorter hospital stays, and improved discharge planning through interprofessional communication and adaptive care planning. However, our unit’s experience indicates that its impact may be limited in certain contexts, such as in the presence of severe delirium, organisational constraints, or limited family engagement, which can hinder the implementation of interdisciplinary strategies. On the other hand, inflexible procedures and hierarchical structures within acute care settings may further constrain these processes, limiting the potential for effective multidisciplinary collaboration (Pradelli et al., 2025).

Future efforts should focus on developing standardized outcome measures that capture not only clinical results, but also the quality of care and the benefits of interdisciplinary collaboration. Measures such as reduction in psychotropic medication use, application of physical restraints, and the assessment of BPSD severity through validated objective instruments, could serve as meaningful benchmarks for assessing and enhancing care integration and team performance.

## Data Availability

The datasets presented in this article are not readily available because of ethical and privacy restrictions. Requests to access the datasets should be directed to the corresponding author.
